# Supplementary material for: Crawling and Gliding: A Computational Model for Shape-Driven Cell Migration
Source: PLoS Comput Biol. 2015 Oct 21;11(10):e1004280. doi: 10.1371/journal.pcbi.1004280 (PMC4619082; doi:10.1371/journal.pcbi.1004280)
Supplement: S1 Code — (ZIP) [file pcbi.1004280.s012.zip › release/tst/doc/html/classCellularPotts-members.html]

Tissue Simulation Toolkit: Member List


Tissue Simulation Toolkit
 0.1.4.1


- Main Page
- Namespaces
- Classes
- Files

- Class List
- Class Hierarchy
- Class Members

CellularPotts Member List

This is the complete list of members for CellularPotts, including all inherited members.

|  |  |  |
| --- | --- | --- |
| AddCell(Dish &beast) | CellularPotts | inline |
| AllocateSigma(int sx, int sy) | CellularPotts | virtual |
| AmoebaeMove(PDE \*PDEfield=0) | CellularPotts |  |
| BaseInitialisation(std::vector< Cell > \*cell) | CellularPotts | protected |
| CellDensity(void) const | CellularPotts |  |
| CellularPotts(std::vector< Cell > \*cells, const int sizex=200, const int sizey=200) | CellularPotts |  |
| CellularPotts(void) | CellularPotts |  |
| Compactness(double \*res\_compactness=0, double \*res\_area=0, double \*res\_cell\_area=0) | CellularPotts |  |
| ConstructInitCells(Dish &beast) | CellularPotts |  |
| DivideCells(void) | CellularPotts | inline |
| DivideCells(std::vector< bool > which\_cells) | CellularPotts |  |
| DrawConvexHull(Graphics \*g, int color=1) | CellularPotts |  |
| FindCellDirections(void) const | CellularPotts |  |
| getCell(int c) | CellularPotts | inline |
| GrowAndDivideCells(int growth\_rate) | CellularPotts |  |
| GrowInCells(int n\_cells, int cellsize, double subfield=1.) | CellularPotts |  |
| GrowInCells(int n\_cells, int cell\_size, int sx, int sy, int offset\_x, int offset\_y) | CellularPotts |  |
| Info class | CellularPotts | friend |
| Mass(void) | CellularPotts | inline |
| MeanCellArea(void) const | CellularPotts |  |
| Morphometry class | CellularPotts | friend |
| Plot(Graphics \*g) | CellularPotts | inline |
| PlotSigma(Graphics \*g, int mag=2) | CellularPotts |  |
| ReadZygotePicture(void) | CellularPotts |  |
| Replace(Graphics \*g) | CellularPotts |  |
| ResetTargetLengths(void) | CellularPotts |  |
| SearchNandPlot(Graphics \*g=0, bool get\_neighbours=true) | CellularPotts |  |
| SearchNeighbours(void) | CellularPotts | inline |
| SetRandomTypes(void) | CellularPotts |  |
| ShowDirections(Graphics &g, const Dir \*celldir) const | CellularPotts |  |
| sigma | CellularPotts | protected |
| Sigma(const int x, const int y) const | CellularPotts | inline |
| sizex | CellularPotts | protected |
| SizeX() const | CellularPotts | inline |
| sizey | CellularPotts | protected |
| SizeY() const | CellularPotts | inline |
| spins\_converted | CellularPotts |  |
| ThrowInCells(int n, int cellsize) | CellularPotts |  |
| Time() const | CellularPotts | inline |
| ZygoteArea() const | CellularPotts | inline |
| ~CellularPotts() | CellularPotts | virtual |


---

Generated on Thu Aug 14 2014 22:04:01 for Tissue Simulation Toolkit by  

 1.8.6
